# Designing Interfaces to Display Sensor Data: A Case Study in the Human-Building Interaction Field Targeting a University Community

**DOI:** 10.3390/s22093361

**Published:** 2022-04-27

**Authors:** Chiara Ceccarini, Silvia Mirri, Catia Prandi

**Affiliations:** 1 Department of Computer Science and Engineering, University of Bologna, 40126 Bologna, Italy; chiara.ceccarini6@unibo.it (C.C.); silvia.mirri@unibo.it (S.M.); 2 ITI/LARSyS, 9020-105 Funchal, Portugal

**Keywords:** sensors data, human-building interaction, smart campus, university community, interactive design cycle, interfaces

## Abstract

The increase of smart buildings with Building Information Modeling (BIM) and Building Management Systems (BMS) has created a large amount of data, including those coming from sensors. These data are intended for monitoring the building conditions by authorized personnel, not being available to all building occupants. In this paper, we evaluate, from a qualitative point of view, if a user interface designed for a specific community can increase occupants’ context-awareness about environmental issues within a building, supporting them to make more informed decisions that best suit their needs. We designed a user interface addressed to the student community of a smart campus, adopting an Iterative Design Cycle methodology, and engaged 48 students by means of structured interviews with the aim of collecting their feedback and conducting a qualitative analysis. The results obtained show the interest of this community in having access to information about the environmental data within smart campus buildings. For example, students were more interested in data about temperature and brightness, rather than humidity. As a further result of this study, we have extrapolated a series of design recommendations to support the creation of map-based user interfaces that we found to be effective in such contexts.

## 1. Introduction

In recent years, technological advancement, especially concerning 5G and advanced Internet of Things (IoT) devices, has increasingly led to the transition from buildings to smart buildings. Following the definition made in [1], when we talk about a Smart Building, we refer to a building capable of adapting (not reacting) to its occupants’ perception, comfort, and satisfaction, while keeping a high level of energy efficiency [2]. The ability to adapt comes from the increase in the number of smart devices and sensors installed inside the building. As a matter of fact, Smart Buildings have a building management system (BMS), a system that is able to monitor, regulate, and keep efficient its subsystems, e.g., the HVAC (Heating, Ventilation & Air Conditioning) system, hot water system, or electrical monitoring system [3]. The data coming from the sensors are intended for monitoring the indoor condition by authorized personnel that can exploit Building Information Modeling (BIM), a 3D digital model of the building that displays information for different kinds of stakeholders (e.g., planners, engineers, or managers) [4]. The BIM can be considered as a “digital twin” of the building exploited during all of the building’s lifetime [5], where the monitoring of the indoor conditions is fundamental for the satisfaction and comfort of the building’s occupants [6].

Such a rich context calls for a specific investigation of the several dynamics that relate to the human, the buildings, and the urban landscape: Human–Building Interaction (HBI) is an interdisciplinary field that combines Human–Computer Interaction (HCI), Ubiquitous Computing, and Architecture and Urban Design [7,8]. Usually, studies in the HBI field exploit the data gathered from the sensors to start a discussion with the occupants, with the final aim of increasing their comfort, but, in the end, the actual data are not available to the people who live or work inside the building. In this context, our assumption is that, if visualized in a clear and intuitive way, the data from sensors can raise the target users’ awareness of sustainability issues [9,10], or can be helpful in communication about comfort and energy use [11,12,13]. Therefore, increasing the awareness of occupants can help them make more informed decisions that best suit their actual needs within the buildings. The state of the art in this specific field shows that some previous work on HBI [7,14] and on how to visualize smart building data [15,16] has already been conducted, with the aim of improving the occupants’ experience while exploiting building sensor data (with specific regard to those collecting environmental data, such as temperature and energy consumption). However, the possibility of increasing occupants’ awareness of environmental conditions by means of specific HBI and data visualization strategies (targeting non-expert occupants) is not yet well investigated.

In this paper, we present our study in the field of HBI. In particular, through an Iterative Design Cycle, we designed an interface addressed to the student community of a smart campus that showed the building data, the sensors, and the environmental data collected through them. We have focused in this study on students since they represent the most significant and populous part of a campus community. Engaging 48 students in structured interviews, we demonstrated the interest of such a community in having this kind of information, and we extrapolated a series of design steps to create such interfaces. It is worth mentioning that this study is HCI-based, within the specific context of a comparison between two different user interface prototypes we have proposed to the involved users. Due to its nature, this study cannot be based on a comparison with similar case studies, even because they are not available in the literature, as we are going to discuss in the rest of the paper. Summing up, the most significant contributions of our study in the investigated field are the following ones:

- a qualitative evaluation of a proposed interactive system and its user interface, in an HBI framework, by engaging participants from the student community;
- identification of a series of design recommendations to create such kinds of user interfaces.

The rest of the paper is organized as follows. Section 2 describes studies that investigate (i) Human-Building Interaction and (ii) Data Visualization of data from the smart building. Inspired by these studies, we exploited an Iterative Design Cycle to design two prototypes, as presented in Section 3. Then, we discuss the results of the evaluation of the two prototypes in Section 6. Then, we discuss the implications, limitations, and future work in Section 7. Finally, Section 8 concludes the paper with some final remarks.

## 2. Related Work

### 2.1. Human-Building Interaction

In recent years, the increment of smart buildings, with advancements in sensing and actuation systems, brought out the need for the HCI community to intervene and involve the user in Human-Building Interaction (HBI) design practices [14]. Hence, recent studies have focused on this interaction to improve or facilitate the lives of smart building occupants [17]. In particular, Finnigan and Clear have analyzed how student occupants experienced spaces, how they evaluate them, and how building management might improve considering the occupants [18]. Their case study was based on a smart university building in the UK equipped with a BMS. They recruited 16 students for “building walks” and a speculative design workshop. Their findings concerning the students’ experience inside a smart building provided insights into the design of building interaction within the university context.

Other examples involve sensors and BMS for starting a dialogue and resolving possible thermal comfort tensions inside shared spaces [19,20]. Clear et al. developed ThermoKiosk, a system composed of survey devices (to express a subjective thermal comfort opinion), digital displays (to see the recorded data), and temperature sensors. This system exploited subjective comfort data to analyze how thermal comfort was perceived by occupants [20]. After three weeks of deployment, their findings from the qualitative studies highlighted how the interaction with the system changed thermal comfort perception. Thermal comfort was also studied in [19], where von Frankenberg presented a four-layer framework that aimed to resolve thermal comfort conflicts inside shared spaces and provide fair decision-making, starting from a recorded event, such as environmental and individual biosignal sensor measurements.

The adoption of an innovative approach with the aim of motivating occupants to consider personal energy usage (having positive effects on their smart environments) is the basis of different studies. In particular, ref. [21] proposed a gamification approach as a novel framework for smart building infrastructure, introducing a strategy that incorporates humans-in-the-loop modeling by creating an interface to allow building managers to interact with occupants and potentially incentivize energy-efficient behavior. Moreover, such an approach has been enriched by deep-learning training, so as to improve estimations of occupant actions toward energy efficiency. Other studies focused their research goals and efforts on defining and adopting simulation strategies to analyze and predict occupants’ behavior and then computing metrics and side effects from smart buildings [22].

Summing up, previous works on HBI has focused on improving the experience of building occupants by exploiting sensor data, especially those which collect data about the temperature, energy usage, and individual (or subjective) data, leaving out the possibility of increasing occupants’ awareness of environmental conditions inside a smart building.

### 2.2. Smart Building Data Visualization

Following the increasing presence of smart buildings, BIM and BMSs have become increasingly relevant for facing the challenge of handling large, complex, and dynamic sensor data. In this context, Data Visualization is effective for the analysis and management of this information. In [16], Ignatov and Gade used a 2D digital map of a building to visualize the BIM sensor data. In particular, they created a visualization showing the dust accumulation in each room, exploiting a color scale from blue (lower) to red (higher) for the different accumulation levels. In contrast, Natephra and Motamedi created a virtual 3D environment enjoyable through a head-mounted display to monitor indoor comfort. They showed, inside the environment, information about temperature, humidity, and light as textual labels close to a relative icon, so as to help with the monitoring of indoor conditions [23]. The BIM visualizations were the subject of the review by Ivson et al. [15]. In their review, they recognized three main types of BIM data visualizations: 2D CAD (e.g., schematic drawings, blueprints, maps), 3D CAD (e.g., actual shapes of physical entities), and charts (e.g., tables, hierarchies, graphs, diagrams, plots). Usually, these visualizations had five different scopes: (i) facility management; (ii) sustainability analysis; (iii) work execution; (iv) work planning; and (v) design review and clash analysis.

To summarize, despite the relevance of the role of occupants in relation to smart buildings, the visualizations that display environmental data from BMSs have a specific objective: monitor, manage or control the building [24,25]. Hence, the challenge is how to design visualizations that target non-expert building occupants to provide clear and meaningful insights that can eventually foster their awareness.

## 3. Materials and Methods

### 3.1. The System

**Figure 1:**
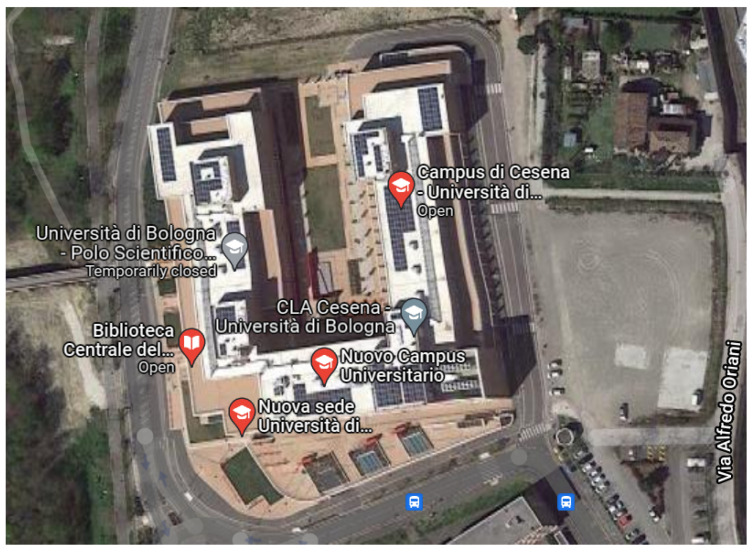
A satellite view of the new Cesena Campus Building.

Our case study focusses on the new Cesena campus building of the University of Bologna, equipped with a sensor infrastructure (BMS) to increase the sustainability of the building. Figure 1 shows a satellite view of the building taken from Google Maps.

**Figure 2:**
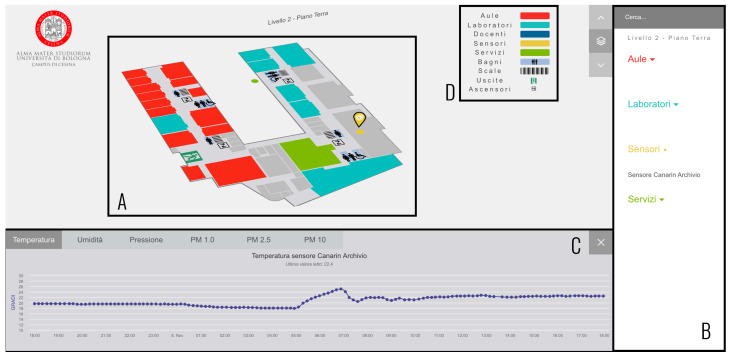
The system interface displayed when a user clicks on a sensor. (**A**) represents the floor where the sensor is located; (**B**) represents the sidebar with all the PoIs listed; (**C**) represents the historical visualization of the temperature data gathered by an indoor sensor; (**D**) represents the legend with all the space typologies (e.g., classrooms, laboratories, offices, bathroom).

We decided to equip this smart building infrastructure with other low-cost sensors for indoor and outdoor measurements, exploiting the Canarin II ([26]) sensor stations, which measured temperature, humidity, air pressure, and air quality in form of PM${}_{1.0}$ (Particulate Matter), PM${}_{2.5}$, and PM${}_{10}$. Therefore, we created a system composed of four layers: (i) a sensor infrastructure, (ii) storage/database management, (iii) a web server, and (iv) a data visualization interface (more details can be found in [9]). For this study, we focused on the data visualization interface and, in particular, on the design of such an interface that provided an interactive way to discover the hyperlocal data generated by the smart campus. This interface was built by exploiting standard web technologies, such as HTML5, CSS3, and JavaScript, and some specific libraries (D3.js (https://d3js.org/, accessed on 15 January 2022) and Chart.js (https://www.chartjs.org/, accessed on 15 January 2022)) to visualize the data as graphs. The interface was used through a browser (Google Chrome), using the kiosk modality available in Windows 10 OS, installed in a public touchscreen display (32${}^{\prime \prime }$ capacitive touch monitor) positioned at the main campus entrance. The interface shows a 2D map of all the floors of the campus, and clicking on one floor will expand it, letting the user explores the spaces (classrooms, labs, offices, libraries, services, canteen) on that specific floor. An example of a floor is visible in Figure 2A, visualizing all the Points of Interest (PoIs) for the campus community, such as classrooms, laboratories, professors’ offices, or facilities. All the PoIs are listed in the sidebar, where a user can also search or filter them through the search bar (Figure 2B). Moreover, a user can explore and visualize real-time and historical data gathered by the sensors infrastructure through an interactive timeline (in Figure 2C) that appears when the relative sensor is clicked (Figure 2). The goal was to make the community aware of the indoor (and outdoor) environmental conditions.

### 3.2. Our Methodology

**Figure 3:**
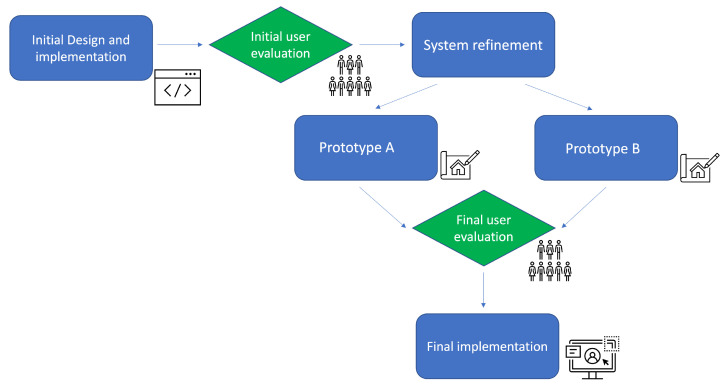
A flowchart showing the stages of the adopted methodology.

After deployment of our system, we were interested in understanding if the data were represented in a clear and intuitive way, so as to eventually foster the community’s awareness of the environmental conditions [27]. Moreover, we wanted to investigate the possibility of including the student community in the design process, since they are the primary target audience of our system and the most significant part of a campus community. To do so and to test the usability of the system, we adopted a methodology based on an Iterative Design Cycle [28] composed of the following main different stages (as depicted in Figure 3):

1. an initial evaluation of the system through structured interviews and related results analysis (as detailed in Section 4);
2. the redesign of the system by the creation of two prototypes (Prototype A and Prototype B) (as detailed in Section 5);
3. a re-evaluation of the systems through the interaction with the prototypes (as detailed in Section 6);
4. the refinement of the initial system based on the new evaluation.

#### Interviews

In both the evaluation sessions (stages 1 and 3 in the above list), we engaged voluntary students in a structured interview. Standing in the proximity of the main entrance, we directly approached random students, asking them to participate in the study. When they accepted, the researcher started the session by explaining the goal of the study, the voluntary nature of the participation, including the possibility to interrupt the interview at any moment, and, finally, she provided the participant with a paper on informative consent, detailing issues about data analysis and storage, in accordance with the European GDPR (General Data Protection Regulation).

The interviews (stages 1 and 3) were conducted by two different facilitators: one was directly involved in the dialog with the interviewees, while the other one annotated the words, gestures, and expressions of the interviewees. All interviewees faced the interview in the same conditions: they stood in front of the kiosk monitor with the same facilitators. Considering the tasks we asked them to perform, all of them started from the system homepage and conducted the requested activities in the same order.

The interviews were structured into four phases:

1. personal questions, (Q1–Q3 in Table 1);
2. questions about the use of our system, (Q4, Q5 in Table 1);
3. two interaction tasks;
4. feedback: general questions on the system, Q6–Q11 in Table 1).

It is worth noticing that the questions in the structured interview (reported in Table 1) were inspired by our previous study [9]. In fact, since we had already evaluated the overall system usability, interactivity, and the level of simplicity to reach the needed information (exploiting quantitative data), in this study, we wanted to focus specifically on qualitative data. In particular, thanks to the structured interviews, we were able to collect qualitative data to understand the critical issues related to the user interface and data visualization discovered while interacting with the system. For this reason, we only needed simple questions to engage the participants and collect feedback and comments, instead of validated scales to measure specific dimensions.

The reason for Q4 was to divide interviewees into two different groups: the “inexperienced” users, in other words, those who had never interacted with the system before, and the “experienced” users, those who had already interacted with it. In fact, at the moment of the evaluation, the application had already been installed for a while in the public kiosk available inside the Cesena campus, and students could freely interact with it. The division into two such groups is relevant in order to evaluate how intuitive the system was for the “inexperienced” users and how much it had impressed the “experienced” ones. Depending on this division, Q5 was different for “experienced” (Q5.a) and “inexperienced” (Q5.b) users.

For the interaction tasks, the thinking-aloud protocol was exploited while testing the system’s usability [29]. The facilitator asked the user to verbally express their thoughts during the interaction with the interface.

## 4. The Initial User Evaluation

We conducted 38 interviews with students frequenting the Cesena campus (I1-I38). The sample of participants was as follows: 8 (21%) female and 30 (79%) male, with ages ranging from 19 to 30, 30 (79%) bachelor students, 7 (18%) master students, and 1 Ph.D. student. The students were mostly from the Computer Science and Engineering area (31); the remaining were enrolled in different degrees, including architecture and biomedical engineering. It is worth mentioning that these percentages reflect the student population structure in the Campus we have used for our case study.

In this initial user evaluation, the interaction tasks were based on the following two scenarios:

1. The first scenario was “As a student, you would like to attend the lesson of the X class held by professor Y”, where X was a lecture taking place that day, and Y was the respective professor’s name. In this case, we wanted the user to find the right classroom.
2. The second scenario was “As a student, you have requested a consultation with Professor Y. The meeting has been confirmed in her office”, where Y was the professor’s name. In this case, we wanted the user to find an office.

It is worth noticing that the users were not explicitly told to find a place, but that the activity to be completed was hidden by describing a general scenario. In this way, we did not influence the interviewee, and we did not help them in the steps to complete the activity. In order to perform a more accurate analysis, all users started from the home screen to perform the two tasks. Such interaction tasks had two purposes. Firstly, they allowed the users to interact in first person with the system and answer the questions in a more concrete and precise way. Secondly, they helped us to see the problems that arose in the usage and interaction.

### Results

14 interviewees (37%) were experienced users (Q4), as they had already interacted with the system: 7 of them out of curiosity; 4 of them while searching a lab, a classroom, or an office; 2 of them to find available classrooms where to study; and 1 to see the arrangement of classrooms and bathrooms (Q5.a). 24 interviewees (73%) were inexperienced users (Q4): 9 of them never approached the system since they did not understand its usefulness or its functionality, 6 of them were not curious about it, 4 of them did not see the monitor at the entrance, 4 already knew where to go, 1 said that it was difficult to use it, and 1 did not interact with it for lack of time (Q5.b). This information allowed us to reflect on three issues: (i) the project was not publicized; (ii) the location of the monitor was not noticeable, perhaps the inclusion of a sign or pictures would have allowed students to notice the monitor; and (iii) there was no guideline detailing how to interact with the system.

All participants were able to complete the interaction tasks. More problems were encountered with the first scenario because many users were not familiar with the system. For the same reason, the duration of the activities changed: the first one lasted from a minimum of one minute to a maximum of three minutes, while the second took less than a minute. These factors led us to think that the system, after only one use, was considered easy to use by the users, and that they were able to understand how to reduce the search time by making fewer steps, thus finding the shortest way to perform the task. After the activities had been carried out and an overview of the system’s functionalities was given, most participants thought it was useful (92%) (Q6).

Concerning Q7, many interviewees suggested having a list of the functionalities on the homepage instead of the building map, as they had not thought it was an interactive system. I1 said “I would like an introductory screen that quickly explains the system’s features”. Regarding the usability, many participants did not notice the search bar, which would have facilitated the two activities. I3 said: “The search should be highlighted better and be more visible, with an icon that makes it more evident”. Moreover, some students suggested adding a way-finding functionality with all indications, such as on Google Maps. For many interviewees, it was important to find spaces (classrooms, library, open spaces) where to study or to wait for the beginning of a new lesson (Q8). I5 said: “It would be useful to include a function that indicates which classrooms are free at the time of the search”, I9 said: “It would be useful to indicate which spaces are dedicated to students”, I24 said: “It would be useful to indicate the hours of the library and if there are places available”. Some participants believed that it was nice to use the system to advertise the events organized by the university. In fact, I31 said: “It would be good to put the events on the screen”. Concerning the sensors (Q9 and Q10), we found two different groups of interviewees. One group was interested in the sensors and their information. For example, I15 stated: “I would like to have a description of the sensors with more detailed information for each of them, with also a global view”. A second group did not notice them or could not figure out what they were or represented.

Finally, 32 students (84%) were interested in having a mobile version to choose their course (to decrease the search time), geolocate the classrooms, and see free spaces. On the contrary, 4 participants claimed that they would not use the mobile version because they are not fond of mobile apps.

## 5. The Prototype Design

After analysis of the interviews, we focused on the sensors, attempting to understand how to improve the visualization of the data collected from them. To do so, we decided to create two different prototypes to establish the preferred one. Three types of data were chosen based on previous interviews, i.e., based on what users expressed they wanted to see and what they felt was more meaningful regarding the sensors. Therefore, it was chosen to represent data regarding temperature, brightness, and humidity. In these prototypes, we placed three images on the right side of the map, referring to the typology of the data collected. We decided to use images familiar to the user (i.e., images used in other applications or objects of common use) so s/he can understand their meanings by simply looking at them. In particular, we selected a sun for the light sensor, a thermostat for the temperature, and a droplet for the humidity.

**Figure 4:**
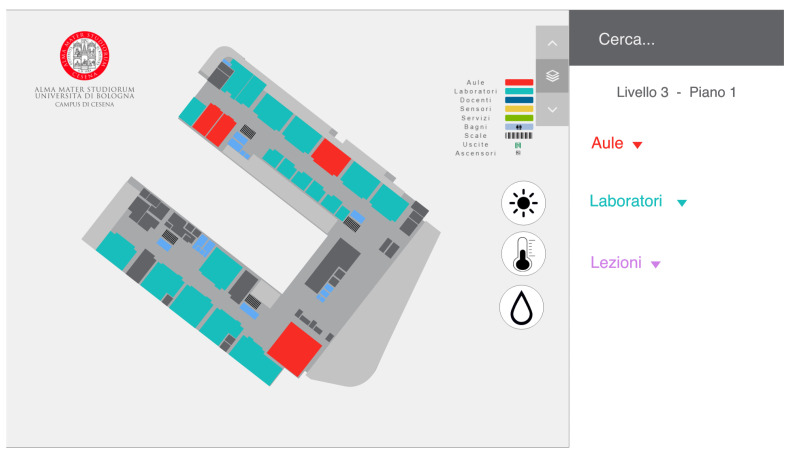
The homepage of the two prototypes with the three icons on the left that showed the three types of sensor data to visualize (brightness, temperature, and humidity).

Both prototypes had the same main screen of the system already deployed to avoid confusion. The only difference was the addition of the three icons on the left that represented the sensors data that could be visualized (Figure 4).

**Figure 5:**
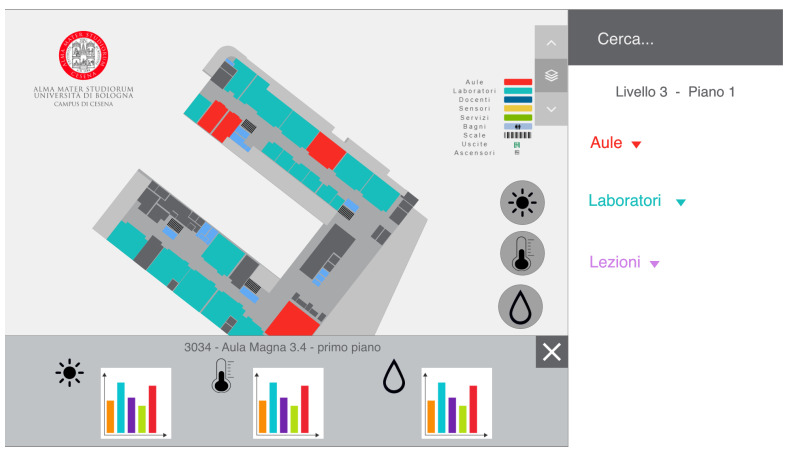
When a space is selected, the graphs for all the data will be displayed.

Moreover, by clicking on each space in both of the prototypes, the user could see more detailed and historical information about the data collected through some graphs, as shown in Figure 5.

### 5.1. First Prototype

**Figure 6:**
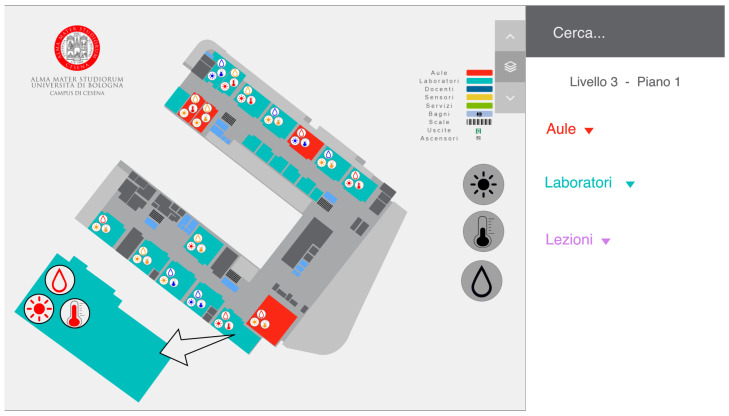
The UI of the first prototype with the three icons on the left selected. The three icons were all selected so each space has inside it the three icons displaying the current value for each sensor, as shown in the zoomed space on the bottom left of the figure.

In the first prototype, we chose to visualize the current data coming from the sensors as a colored icon within the space where they were located (lab or classroom), as shown in Figure 6. By default, the sensors were not displayed but appeared when the user clicked on the relevant side icon. In this way, the user could choose how many sensors to visualize at the same time and have a general overview of all the floors. When an icon was selected, its color would change into gray to make the selection clearer. Conversely, the color of the sensors’ icons in each space would change based on the data collected. In particular, it would be red if the value was high, orange for a medium value, and blue for a low value. We chose these three colors as they are familiar to the user and usually exploited to indicate high or low values.

### 5.2. Second Prototype

**Figure 7:**
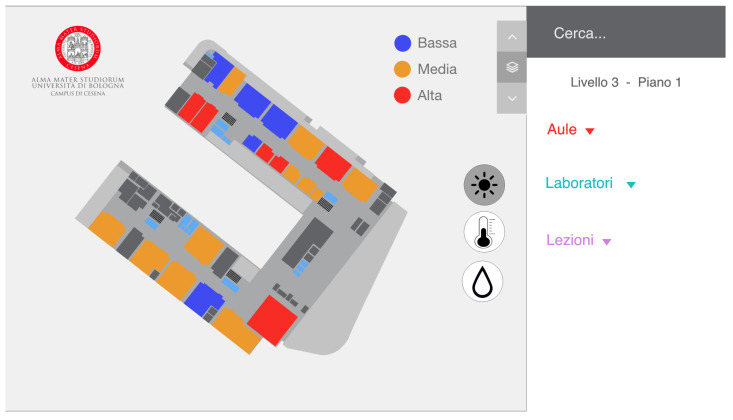
The UI of the second prototype with the brightness icons selected. Each space is colored based on the current data gathered by the brightness sensors. A legend for the color is also displayed to help the users and avoid confusion.

In the second prototype, we chose to color the spaces according to the data collected by the sensors. For example, if the user clicks on the brightness button, each classroom will change color according to the values of that sensor (Figure 7). The choice of colors is the same as in the first prototype. In this case, we decided to include a color legend to avoid confusing a user with the change of color. In this prototype, there was no possibility of seeing the data of all the sensors at the same time, so there was no general overview.

## 6. The Final User Evaluation

As already mentioned in Section 3.2, to analyze the usability and intuitiveness of the two prototypes, we conducted another series of interviews targeting students on campus. Therefore, the purpose of these interviews was to find the best solution between the two prototypes and to draw some conclusions on how the sensor data should be displayed. The interview was carried out by the same two facilitators of the previous interviews. The use of the same facilitators, as well as for organizational reasons, brought three advantages: (i) they already knew the difficulties encountered by the users in the previous interviews, (ii) they were familiar with this mode of interviewing, and (iii) in case a user who had already done the first interview came back, s/he would have felt at ease sooner, having already created a relationship of trust with them.

The protocol adopted was the same. The only difference was that the evaluation, this time, was focused on the two interactive prototypes instead of the whole system. Nonetheless, we visualized the interactive prototype on the same kiosk, so as to not affect users’ confidence in interacting with the system.

In this evaluation, we only defined one interaction task, and we asked participants to repeat it for both the prototypes. The interaction task was: “As a student, you would like to know the indoor conditions inside classroom X”, where X was a classroom occupied by a lecture taking place that day.

### 6.1. Results

We conducted ten interviews with students frequenting the Cesena campus (I1–I10). The participants’ sample was composed as follows: 3 (30%) female and 7 (70%) male, with ages ranging from 20 to 36; 4 (40%) bachelor students, and 6 (60%) master students. In total, 8 interviewees were Computer Science students, 1 was a biomedical engineering student, and 1 a psychology student. It is worth noting that, at this stage of the study, we aimed to collect data devoted to a qualitative study. Involving 10 participants does not give us significant results from a statistical point of view, but they provide precious insights for a qualitative analysis of the obtained data. In fact, according to the literature [30], involving 5 users in testing a user interface and its interaction mechanism would result in the identification of an average number of interaction problems that is equal to 85% of all the problems in that user interface, and 100% of the problems are identified on average by engaging 15 users. Hence, 10 participants would detect the most significant issues with the user interface analyzed.

8 students had already interacted with the system (Q4), but half of them had never noticed the sensors. 7 of the interviewees thought it was useful to have information about environmental conditions collected by sensors in the spaces (Q10).

In general, the interviewees wanted to see information about the temperature (to find the room with the best temperature to stay inside) and light (to see the empty spaces). They weren’t interested in the PM, as some of them didn’t understand its meaning. Moreover, half of them (5) asked for a “presence sensor” to get data about the number of people inside a class/lab.

The prototypes were evaluated based on two dimensions: intuitiveness and satisfaction. The first one indicated that the colors, icons, and interface were easy to understand and use. Meanwhile, by satisfaction, we mean what impression the prototype left on the users, what their first impression was, if they liked the graphics, and all those feelings elicited in the user when using the prototypes. After analyzing and coding the interviews and the observations and field notes taken during the sessions, we can state that the first prototype was intuitive for 90% of the respondents, even if it did not completely satisfy all users (40% of them were not satisfied). The second prototype was both intuitive and satisfactory for 70% of the interviewees. Even if this one was initially less intuitive, it brought more satisfaction than the other.

For both of the prototypes, the students liked having the current data but thought that the visualization of historical data could be avoided.

Finally, from the interviews and the field notes, it emerged that 70% of the respondents preferred the second prototype. Even if they liked the first prototype because it had a global visualization of all three sensors, it brought several doubts about how to manage the space on the map. In fact, I4 said: “The visualization of all the sensors together is useful, but if there are more sensors, it would be difficult to read it because of the limited space”. Interestingly, I5 also said: “The second prototype might interest a general user more than a technician (the other prototype could be better for a technician) because this one is more intuitive.” Concerning the intuitiveness of the two prototypes, there is no clear differentiation, but the satisfaction left by these prototypes was greater in the second one. In fact, I8 said: ”This prototype (referred to the second one) is more beautiful, but, obviously, it is not possible to simultaneously visualize all three sensors on the global map. Although the first one is more intuitive, I like this one more, also because it gives me a rough outline of what I am looking for”; also I1: “This prototype is easier. The legend is useful for understanding what the colors refer to. I prefer this one because the colors have more impact than the other”.

The choice of the preferred prototype was also influenced by their ability to process information related to the sensor data. In this way, the final choice made it possible to deal also with the issue of cognitive overload.

### 6.2. Final Implementation

**Figure 8:**
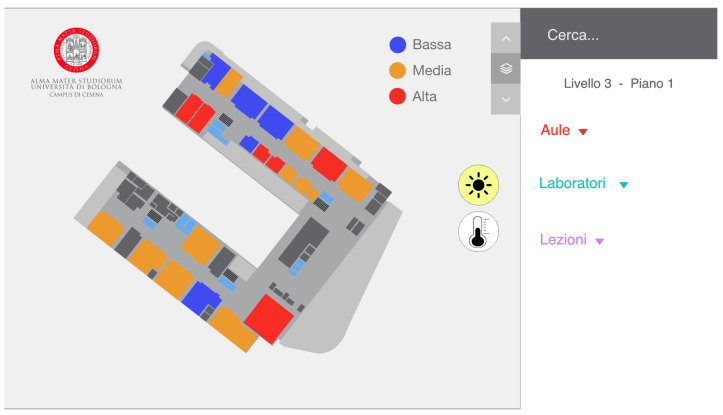
The UI of the final version created after the interviews starting from the second prototype. We kept the legend, and the icons for brightness and temperature, and we changed the color for the selected sensor.

Based on the considerations made after the interviews, the second prototype was chosen for the final version of the system. However, based on the feedback received, some modifications concerning the icons and the colors were implemented (as depicted in Figure 8). As a matter of fact, 80% of the respondents immediately figured out what the icons represented, although not all guessed the meaning of the colors. For this reason, in the final prototype, it was decided to keep the same icons and maintain the legend to indicate the meaning of the colors. In fact, I2 says: “Thanks to the legend of the second prototype, I was able to understand immediately what the colors were referring to, something that I had not immediately understood in the first prototype”. I3 also expressed a consideration about the icon buttons: “The only issue with the icon buttons is the gray color when selected. In fact, you usually use that color to indicate that something is disabled. This could be confusing for the user”. For this reason, in the final version, we decided to change the color for selecting and deselecting the icon buttons, as shown in Figure 8. This choice was also justified by the intention of limiting the cognitive overload of the users. For the same reason, we removed information considered unnecessary by the students, e.g., we eliminated the data about humidity.

## 7. Discussion, Final Remarks and Limitations

In this study, we exploited, as a case study, an interactive web-based system designed with the primary goal to help students and external collaborators to easily locate a classroom/lab/office in the new Cesena Campus building. We then decided to visualize data related to environmental conditions in order to increase occupants’ awareness of environmental conditions inside a smart building. In fact, according to the literature analyzed, two main research challenges need to be addressed in this scenario, where we believe awareness is a crucial dimension to consider. Firstly, several studies about building data visualization used environmental conditions data only for monitoring, managing, or control, without taking into account the non-expert building occupants’ needs and awareness. Secondly, if focusing on the area of HBI, previous studies have focused on improving the experience of building occupants by exploiting sensor data, especially those collecting data about temperature, and energy usage, leaving out the possibility of increasing occupants’ awareness of environmental conditions. To fill these two gaps, we performed a study engaging users (non-expert building occupants) in the design of a system able to visualize sensed data and increase the users’ awareness. To this end, we initially evaluated the system through structured interviews, and then we revised the map-based interface and prototyped two different versions, integrating the users’ comments and suggestions. Finally, users were engaged in the re-evaluation of the systems. This allowed us to refine the system based on the new insights and implement a final version.

All things considered, we can claim that this study has three main novel aspects. First, we exploited an iterative design cycle in a novel domain, that is HBI, actively engaging non-expert building occupants in the process (instead of expert users). Second, we translated the particular insights collected directly involving users into design recommendations. Third, with this work, we intended to fill the gap related to data visualization in the BIM context.

From a practical perspective, we envision the possibility of using the system not solely for the initial purpose of finding lecture halls or faculty offices, but also for increasing awareness about the building’s environmental conditions and, eventually, about sustainability as a human value. In fact, Friedman defines the term human value as “what a person or group of people consider important in life” [31]. This broad notion is accompanied by a non-exclusive list of specific values named Human Welfare, Ownership and Property, Privacy, Freedom from Bias, Universal Usability, Trust, Autonomy, Informed Consent, Accountability, Courtesy, Identity, Calmness, and Environmental Sustainability [31]. In this study, we primarily focus on the last one: Environmental Sustainability.

However, including information about the current temperature and light can help students to choose the best space for where to study or work in a group (i.e., a warmer or cooler classroom or an empty one where the lights are off). As a side effect, this will also ensure that students will not stop using the system once they become aware of classroom/lab locations, engaging students over time. In terms of building environmental conditions, engaging the students in the interviews made us realize that the sensors most appreciated for increasing awareness were temperature and brightness (which led to us removing humidity). Moreover, it is an interesting fact that most of the students pointed out the necessity of having a sensor for counting people inside each space. Finally, all the students also remarked on the importance of having a clear legend, as it helped them or confirmed what they thought about the meaning of the color. This last result is not surprising since it confirms one of the 10 well-known usability heuristics by Jakob Nielsen: “recognition rather than recall” [32]. This heuristic states that: “Minimize the user’s memory load by making elements, actions, and options visible. The user should not have to remember information from one part of the interface to another. Information required to use the design (e.g., field labels or menu items) should be visible or easily retrievable when needed” [32].

From a framework perspective, we can draw some conclusions regarding the design of interfaces that aim to display indoor environmental conditions, using sensors installed inside public and shareable spaces, such as a smart campus. Such data are intended to be provided to a community of non-experts. These conclusions can be translated into a series of recommendations for designing a map-based interface:

1. create a visualization designed for considering specific target communities (students versus staff);
2. understand which sensors to display: before starting the design, it is essential to understand what types of sensors you have available and which are of interest to the target communities to create prototypes with only the necessary information;
3. provide a global map view of a single sensor at a time: this avoids cognitive overload for non-expert users;
4. choose between current data against historical data: avoid adding information that is of no interest to the target communities (in our case study, the historical data were considered useless to the students);
5. choose the right icons for the sensors: icons are more immediate but not always clear without an explanation;
6. choose colors for the data with a clear meaning (i.e., warm and cold colors) and use colors to color the entire space, to give an overall idea of the current situation;
7. use a legend: even if the icon or color seems clear, a visible legend is necessary;
8. add the name of spaces (if possible): not necessarily all users understand which space is which only by looking at the map, so if space allows, add the name.

Our work has two limitations that call for future work. First, the number of students interviewed was limited. This was not a problem for the usability test, as Nielsen and Molich demonstrated that 50% of the major usability problems can be detected with only 3 users [33]. With 5 users, 90% of the most frequent usability problems can be detected. By most frequent problems, we mean those problems that occur with a frequency of at least 31% (which is an average problem frequency) [30]. As the number of participants increases, the percentage of problems with that frequency slightly increases, because each new participant identifies problems already encountered by previous participants. However, the addition of participants also increases the probability of detecting problems with lower frequencies, which can be desirable or even significant. Future studies should include larger sample sizes to achieve more accurate findings. Nonetheless, having analyzed the obtained results in detail, we can affirm that the data provide valuable information, and such insights can be useful for developing cumulative knowledge [34].

Second, in our study, we only considered the student community, as they were the ones who most interacted with the monitor at the entrance. Replicating the study by integrating other communities, such as technical-administrative staff or faculty, could lead to new insights into the project, as they may be interested in aspects not yet analyzed. As one interviewee suggested, the choice of the prototype could be different for technical staff, implicating that the same data or info could benefit various communities if visualized in different ways [35].

## 8. Conclusions

In this paper, we have presented a study we conducted in the Human–Building Interaction field. In particular, through an Iterative Design Cycle approach, we designed an interface addressed to the student community of a smart campus that showed different environmental data. The data were related to the building status and the condition of its rooms and services.

In the study presented, we have engaged 48 students in structured interviews to assess a qualitative evaluation of map-based user interfaces specifically designed and developed for increasing the awareness of smart building occupants in terms of indoor environmental issues, with the final goal of improving conditions within a smart campus. After having analyzed the results obtained, we can confirm the interest of such a community in having this kind of information, and we have identified a series of design steps to create such interfaces. The paper presents a discussion of the findings gathered through the involvement of students as target users, since they are the most representative members of the campus community. Limitations of the whole study and of the approach adopted were also illustrated.

In future work, participatory design and co-design could be exploited to bring interesting insights into the design of data visualization in the BIM context, to increase the students’ awareness. Moreover, in this paper, we have considered a case study in the HBI context, but environmental data can also be useful to users in outdoor contexts. An example would be the visualization of air pollution levels to advise users about streets where they would be less exposed. Similar scenarios should be investigated in future work.

## Figures and Tables

**Table 1:** Questions asked during the interviews.

ID	Questions
Q1	How old are you?
Q2	To which gender identity do you most identify?
Q3	What is your course of study?
Q4	Have you ever interacted with the system?
Q5.a (experienced)	Why did you interact with the system? What did you look for?
Q5.b (inexperienced)	Why have you never interacted with the system?
Conduction of the two Interaction Tasks	
Q6	Do you think the system is useful?
Q7	What would you change in the system?
Q8	What information would you be interested in?
Q9	Have you noticed the sensors?
Q10	Do you think they are useful?

## Data Availability

Data sharing not applicable No new data were created or analyzed in this study. Data sharing is not applicable for this article.
